# Supplementary material for: Photosensitizer Activation Drives Apoptosis by Interorganellar Ca2+ Transfer and Superoxide Production in Bystander Cancer Cells
Source: Cells. 2019 Sep 29;8(10):1175. doi: 10.3390/cells8101175 (PMC6829494; doi:10.3390/cells8101175)
Supplement: Supplementary file 1 [file cells-08-01175-s001.zip › SupplementaryFilesRevised/FigureDataSourceWithDescriptions/Data Source Description.docx]

**Data source files – names, description, corresponding figures**

| **File Name** | **Description of the content** | **Corresponding Figure** |
| --- | --- | --- |
| Data_Figure2.xlsx | Original cytosolic Ca^2+^ signals (Fluo-4) in 2 mM and 0 mM extracellular Ca^2+^ and averages after bleaching correction from  Worksheet 1: irradiated cells  Worksheet 2: first order bystander cells  Worksheet 3: second order bystander cells  Worksheet 4: third order bystander cells  Worksheet 5: fourth order bystander cells  Worksheet 6: fifth order bystander cells  Worksheet 7: Mean peak amplitudes of signals  Worksheet 8: Mean time-to-half-peak of signals and mean bystander order distance from the irradiated cell (used to calculate Ca^2+^ wave propagation speed) | Figure 2 - Panel (a)  Figure 2 - Panel (b)  Figure 2 - Panel (c) |
| Data_Figure3.xlsx | Worksheet 1: Original R-CEPIA1er Ca^2+^ signals from subcellular ROIs  Worksheet 2: Original R-CEPIA1er Ca^2+^ signals at high, medium, low irradiation fluencies (used to calculate Ca^2+^ release rates); control traces  Worksheet 3: Averages of data in worksheet 2 after bleaching correction  Worksheet 4: Release rates and p-values in ANOVA test computed from data in worksheet 2 | Figure 3 - Panel (a)  Figure 3 - Panel (b)  Figure 3 - Panel (c) |
| Data_Figure5.xlsx | Original mitochondrial Ca^2+^ signals (CEPIA2mt) from  Worksheet 1: irradiated cells  Worksheet 2: first order bystander cells  Worksheet 3: second order bystander cells  Worksheet 4: third order bystander cells  Worksheet 5: fourth order bystander cells  Worksheet 6: averages of data in previous worksheets after bleaching correction, control trace | Figure 5 |
| Data_Figure6.xlsx | Worksheet 1: Original G-CEPIA1er, CEPIA2mt and MitoSOX Red signals in irradiated cells in the presence and in the absence of Thapsigargin  Worksheet 2: averages of data in worksheet 1 after bleaching correction  Worksheet 3: Original MitoSOX Red signals (used to calculate time derivatives) in the presence and in the absence of Thapsigargin from first order bystander cells  Worksheet 4: second order bystander cells  Worksheet 5: third order bystander cells  Worksheet 6: fourth order bystander cells  Worksheet 7: Area subtended by the time derivative of MitoSOX signals at each order, averages and p-values in ANOVA test | Figure 6 - Panel (a), (b)  Figure 6 - Panel (b), (c)  Figure 6 - Panel (c) |
| Data_Figure7.xlsx | Worksheet 1: Original hydrogen peroxide (HyPerRed) signals from irradiated cells, average after bleaching correction, control trace  Worksheet 2: Original hydrogen peroxide (HyPerRed) signals from bystander cells | Figure 7 - Panel (a)  Figure 7 - Panel (b) |
| Data_FigureS2.xlsx | Single-cell fluorescence of Caspase-3 biosensor after focal irradiation normalized to pre-stimulus value in bystander cells from the 1^st^ to the 4^th^  Worksheet 1: experiment 1  Worksheet 2: experiment 2  Worksheet 3: experiment 3  Worksheet 4: control experiment in the absence of PS | Figure S2 |
