# Supplementary material for: Photosensitizer Activation Drives Apoptosis by Interorganellar Ca2+ Transfer and Superoxide Production in Bystander Cancer Cells
Source: Cells. 2019 Sep 29;8(10):1175. doi: 10.3390/cells8101175 (PMC6829494; doi:10.3390/cells8101175)
Supplement: Supplementary file 1 [file cells-08-01175-s001.zip › SupplementaryFilesRevised/SupplementaryFigureFiles/Supplementary Figures with Captions.docx]

**Supplementary Figures**


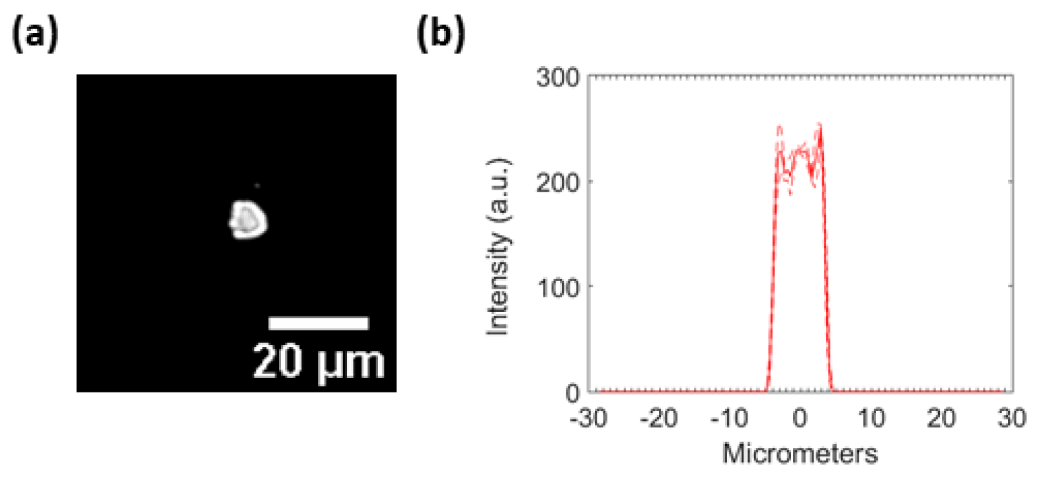


**Figure S1. Photoactivation area.** (a) Image of the irradiated area obtained by photo-ablating a thin black ink film deposited over a microscope glass slide. (b) Average intensity profile ± s.e.m. computed along the directions of n = 3 spot diameters.


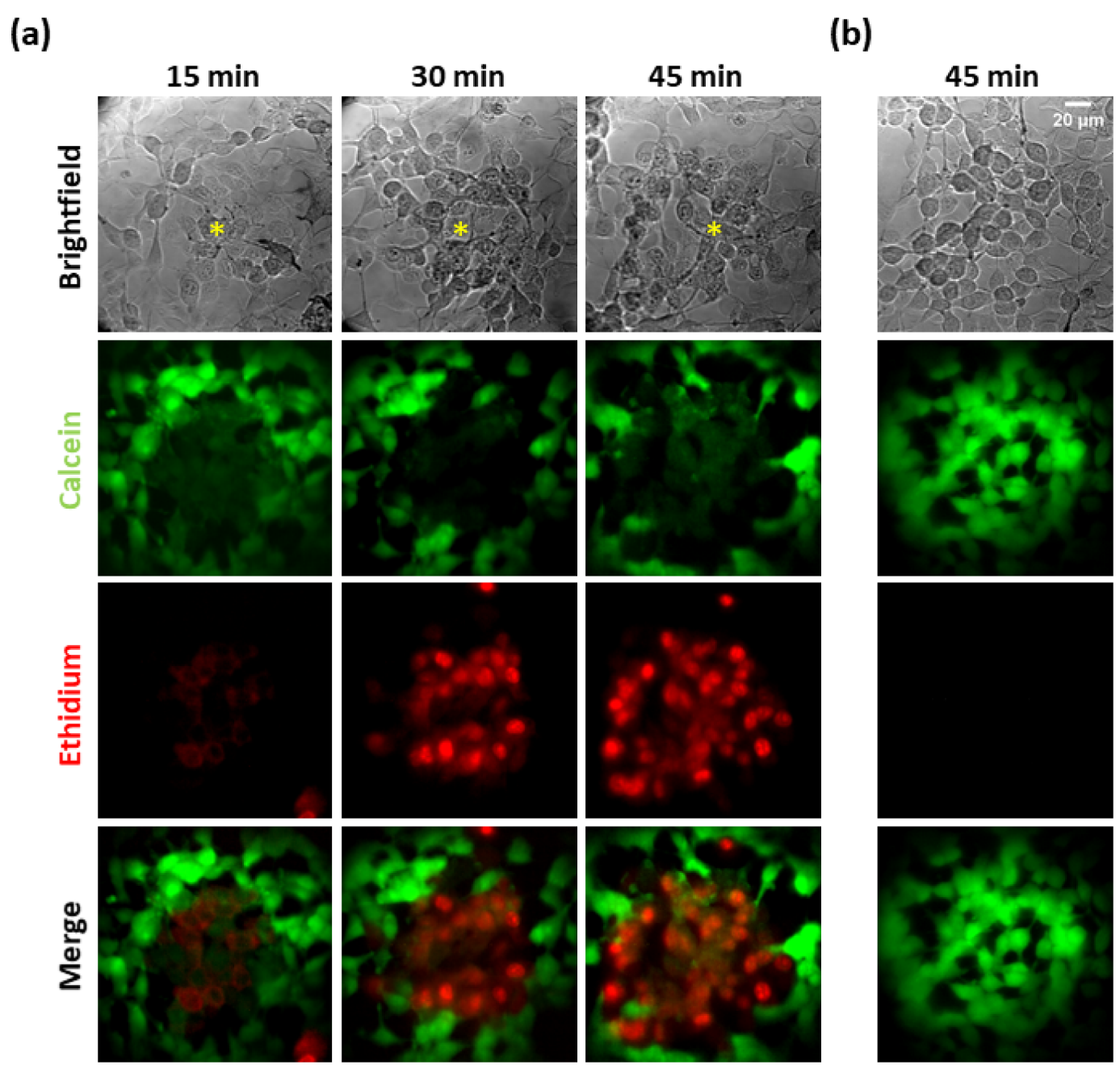


**Figure S2. Cell death process in PS-loaded B16-F10 cell cultures exposed single-cell irradiation.** (a) To generate this figure, a colorimetric live/dead assay was performed on different cultures at different time points after focal irradiation under standard stimulation conditions (see Material and Methods, Section 2.2). Green fluorescence emission corresponds to Calcein AM, that labelled live cells with active esterases; red fluorescence emission indicates Ethidium homodimer-1 (Ethidium), which penetrated the damaged plasma membrane of dying or dead cells. In each image, the irradiated cell is marked with a yellow asterisk. Time *t* = 0 corresponds to the end of photostimulation. (b) Control experiment performed in a PS-loaded culture not exposed to focal laser irradiation.


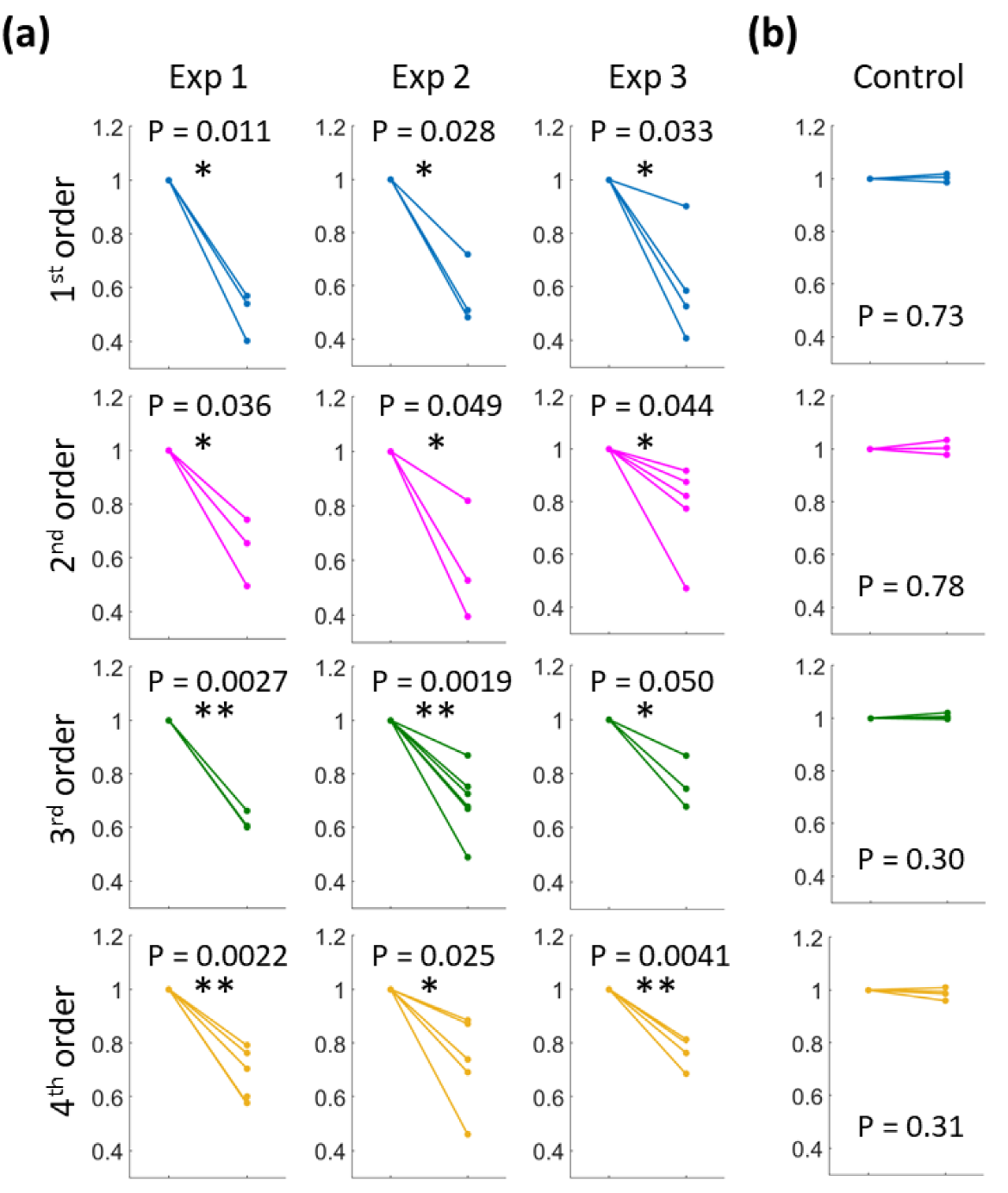


**Figure S3. Focal PS excitation triggers Caspase-3 activation in the irradiated and bystander cells.** (a) Data refer to n = 3 independent experiments (Exp) in PS-loaded B16-F10 bystander cells from the 1^st^ to the 4^th^ order expressing GANLS-DEVD-BNES (a green fluorescent biosensor selective for caspase-3 activity; caspase activation causes a decrease in the biosensor fluorescence emission). Fluorescence signals were acquired before and after focal irradiation under standard stimulation conditions (see Material and Methods, Section 2.2), spatially averaged over each cell and normalized to pre-stimulus level. (b) Representative control experiment conducted in the absence of PS. In (a) and (b), p-values (P) were computed using the paired sample t-test.

**
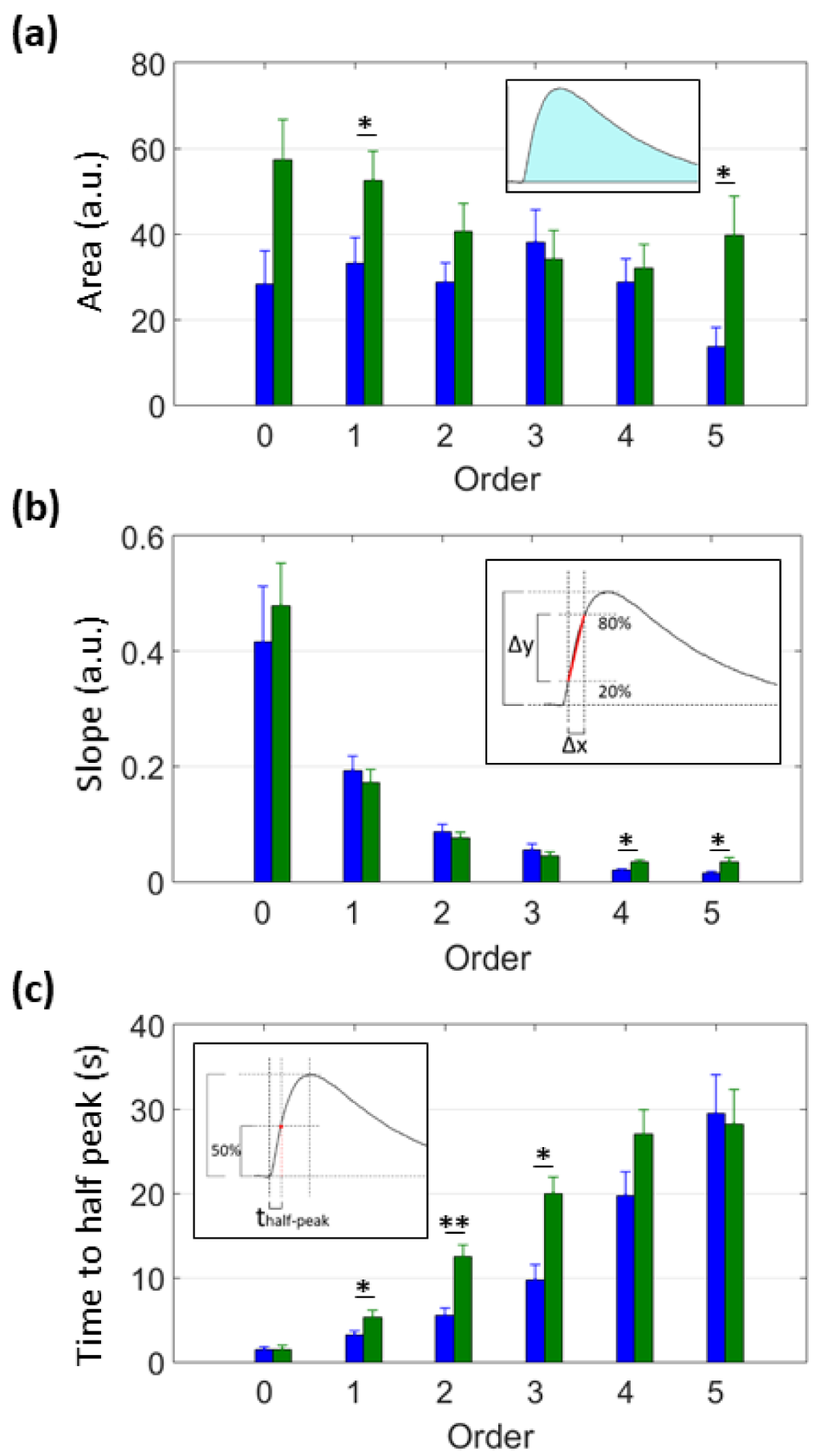
**

**Figure S4. Analysis of cytosolic Ca^2+^ signals triggered in bystander cells following focal PS activation in the presence or in the absence of extracellular Ca^2+^.** Cytosolic Ca^2+^ was monitored with Fluo-4 AM (a Ca^2+^-selective fluorescent dye). Fluorescence signals were acquired before and after focal irradiation under standard stimulation conditions (see Material and Methods, Section 2.2). Mean Ca^2+^ responses were computed for the irradiated cell (order 0) and for each order of bystander cells in the field of view (see **Figure 2**). Each histogram shows the signal parameters identified in the corresponding inset, in 2 mM [Ca^2+^]_e_ (blue bars) and in 0 mM [Ca^2+^]_e_ (green bars): (a) area subtended by the curve, computed within the stimulation time interval (100 s); (b) curve slope, defined as the angular coefficient of the interpolating straight line between 20 % and 80 % of the curve peak amplitude; (c) time to half peak. p-values (P) were computed by ANOVA (*, P < 0.05; **, P < 0.01).


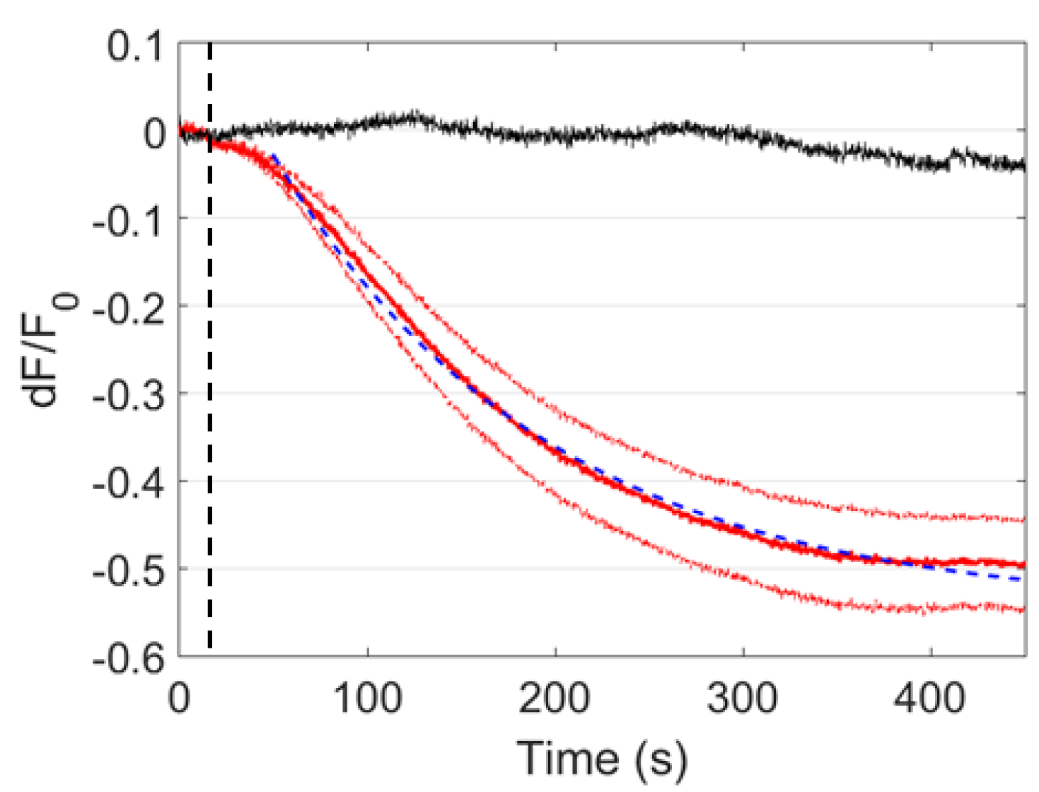


**Figure S5. Kinetics of ER Ca^2+^ store empting by Thapsigargin (Tg).** B16-F10 cells expressing G-CEPIA1er, a green fluorescent Ca^2+^ biosensor targeted to the ER, were superfused with Tg (3 µM). The vertical dashed line marks the onset of Tg application. The G-CEPIA1er mean signal (solid trace) ± s.e.m. (dashed traces) were computed from m = 6 traces in n = 4 independent experiments. The blue dashed line represents exponential curve fitting (performed on single traces and averaged) to obtain the time constant of ER empting (τ = 206 ± 58 s). Single-cell *dF*/*F*_0_ fluorescence traces were computed as average pixel signals within ROIs contouring the whole cell area. The black trace is a representative signal obtained in a control experiment in the absence of Tg.
