# Supplementary material for: Photosensitizer Activation Drives Apoptosis by Interorganellar Ca2+ Transfer and Superoxide Production in Bystander Cancer Cells
Source: Cells. 2019 Sep 29;8(10):1175. doi: 10.3390/cells8101175 (PMC6829494; doi:10.3390/cells8101175)
Supplement: Supplementary file 1 [file cells-08-01175-s001.zip › SupplementaryFilesRevised/SupplementaryVideoFiles/Supplementary Video Descriptions.docx]

**Supplementary Video Descriptions**


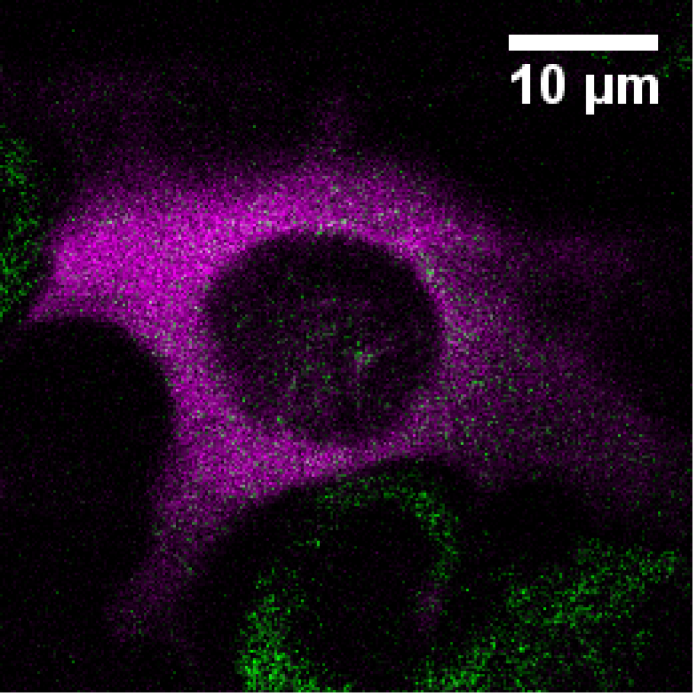


**Video V1. Simultaneous two-color confocal multiphoton imaging of interorganellar Ca^2+^ transfer following PS activation.** A PS-loaded B16-F10 cell co-expressing R-CEPIA1er (a red fluorescent Ca^2+^ biosensor, here shown in purple colour, targeted to the ER) and CEPIA2mt (a green fluorescent Ca^2+^ biosensor targeted to mitochondria) was photostimulated under low irradiance conditions (~7∙10^3^ mW/cm^2^). Images were acquired at 3 Hz for 70 s and played back in the video at 50 Hz.

**
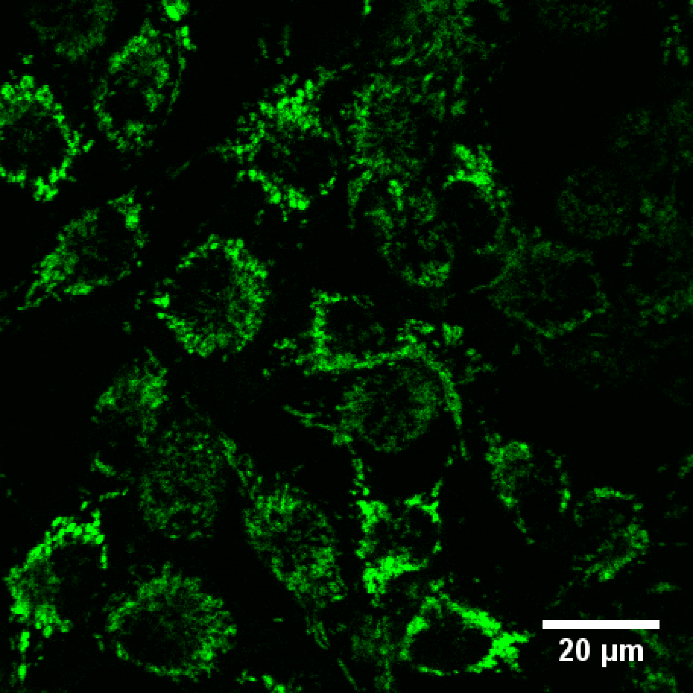
**

**Video V2. Propagation of intercellular mitochondrial Ca^2+^ wave following focal PS activation.** A PS-loaded B16-F10 cell culture expressing CEPIA2mt (a green fluorescent Ca^2+^ biosensor targeted to mitochondria) was focally irradiated under standard stimulation condition (see Material and Methods, Section 2.2). Images were acquired at 5 Hz for 80 s and played back in the video at 250 Hz.

**
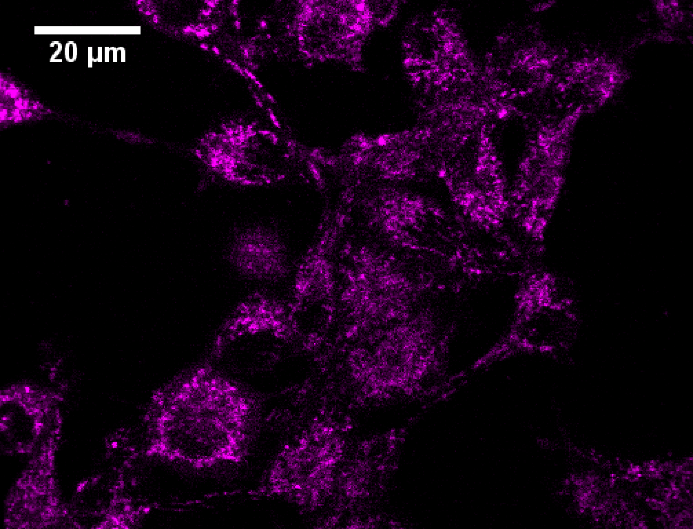
**

**Video V3. Mitochondrial O_2_^¯¯^∙ production in bystander cells following focal PS activation.** A B16-F10 cell culture co-loaded with the PS and MitoSOX Red (a red fluorescent indicator selective for mitochondrial O_2_^¯¯^∙) was focally irradiated under standard stimulation condition (see Material and Methods, Section 2.2). Images were acquired at 5 Hz for 80 s and played back in the video at 250 Hz.
