# Supplementary figures and images for: Photosensitizer Activation Drives Apoptosis by Interorganellar Ca2+ Transfer and Superoxide Production in Bystander Cancer Cells
Source: Cells. 2019 Sep 29;8(10):1175. doi: 10.3390/cells8101175 (PMC6829494; doi:10.3390/cells8101175)

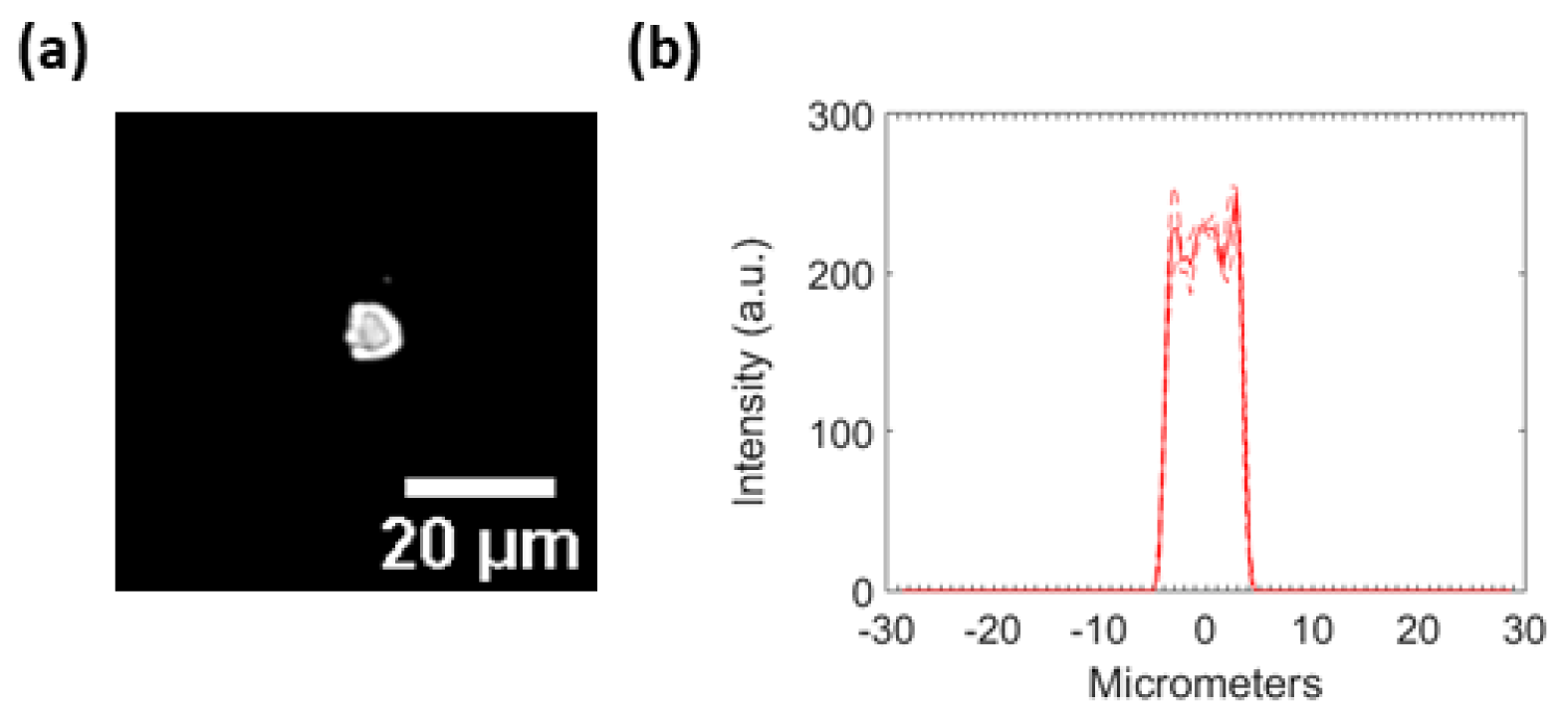

Supplement: Supplementary file 1 [file cells-08-01175-s001.zip › SupplementaryFilesRevised/SupplementaryFigureFiles/FigS1.tif]

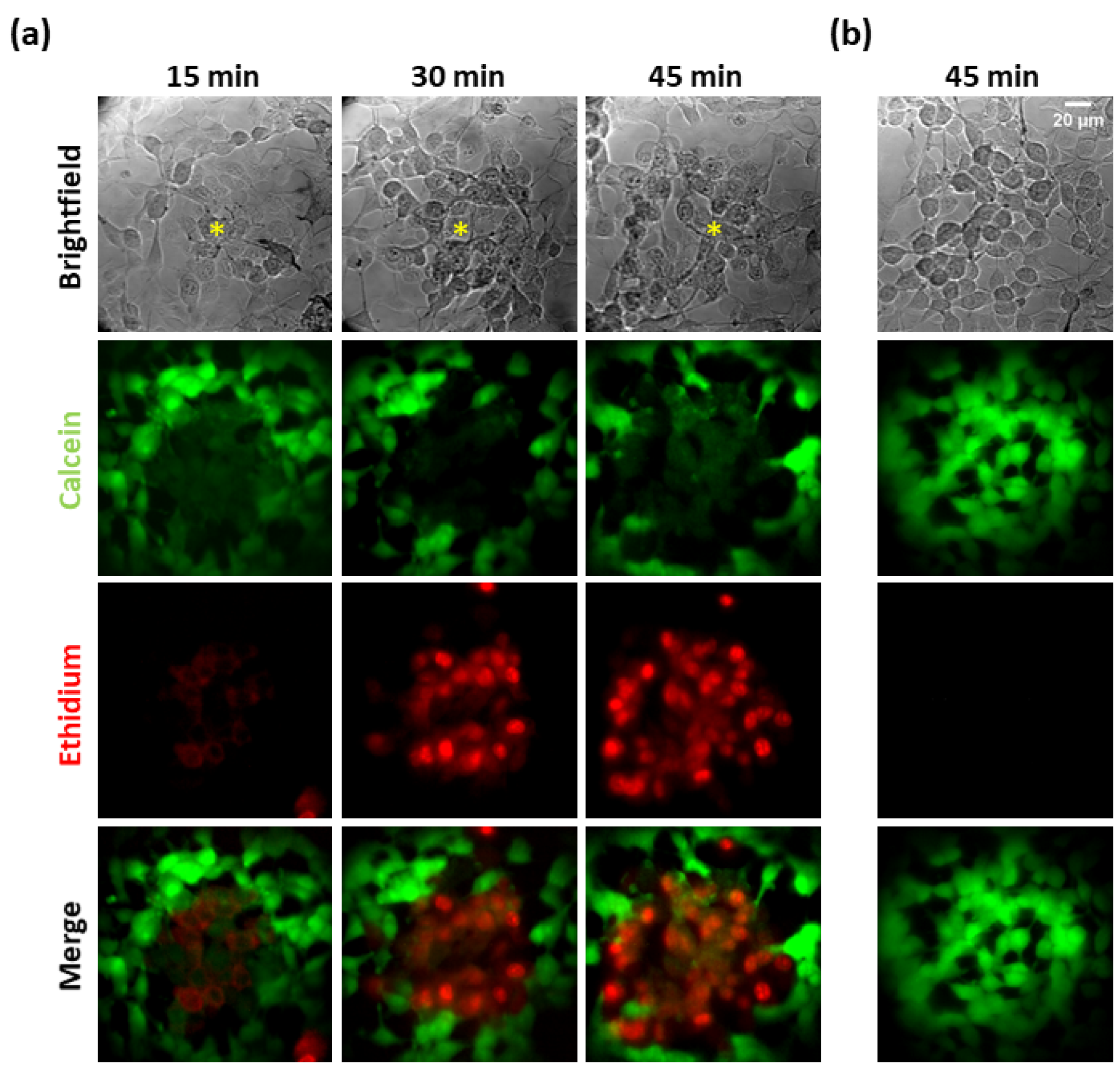

Supplement: Supplementary file 1 [file cells-08-01175-s001.zip › SupplementaryFilesRevised/SupplementaryFigureFiles/FigS2.tif]

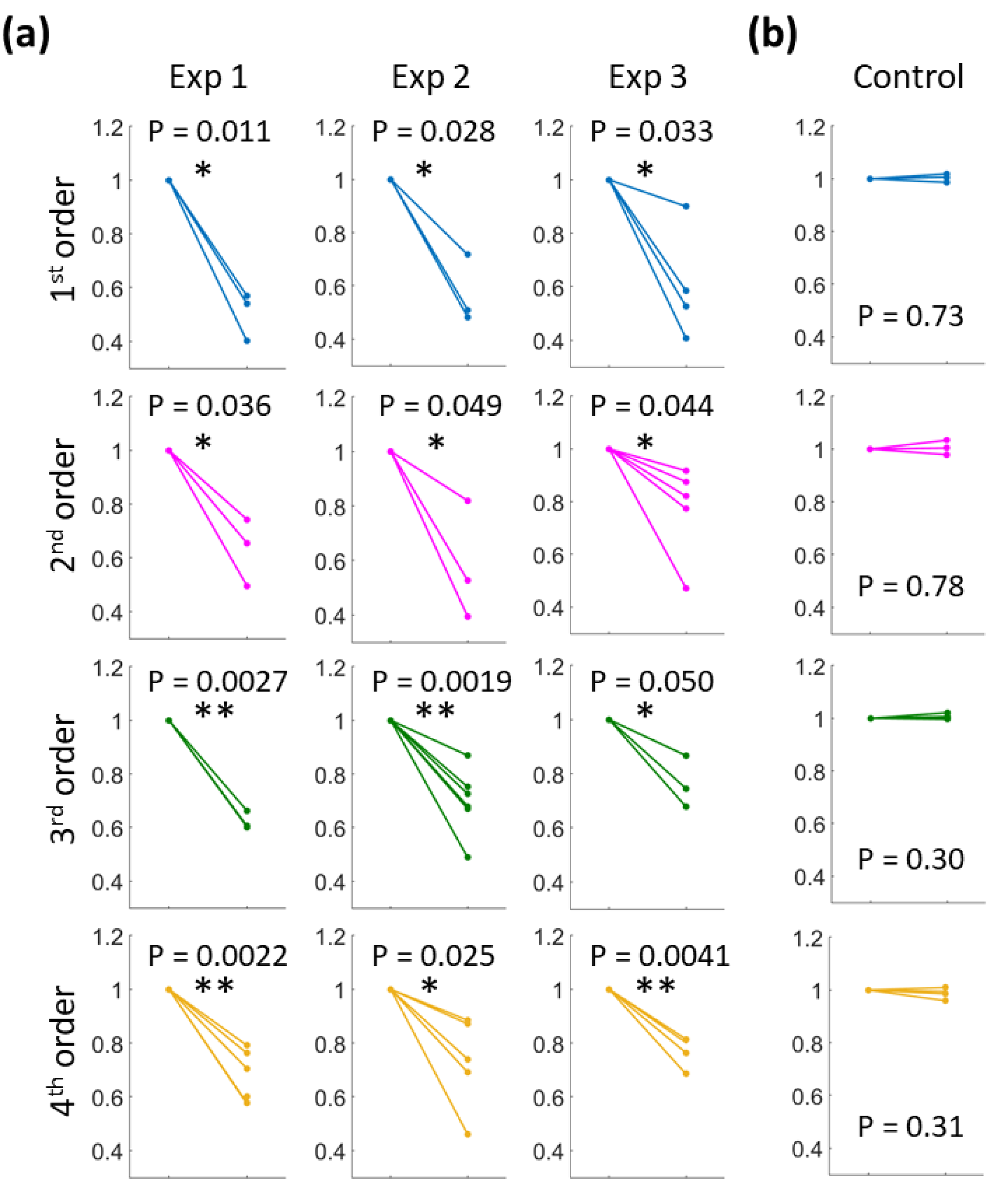

Supplement: Supplementary file 1 [file cells-08-01175-s001.zip › SupplementaryFilesRevised/SupplementaryFigureFiles/FigS3.tif]

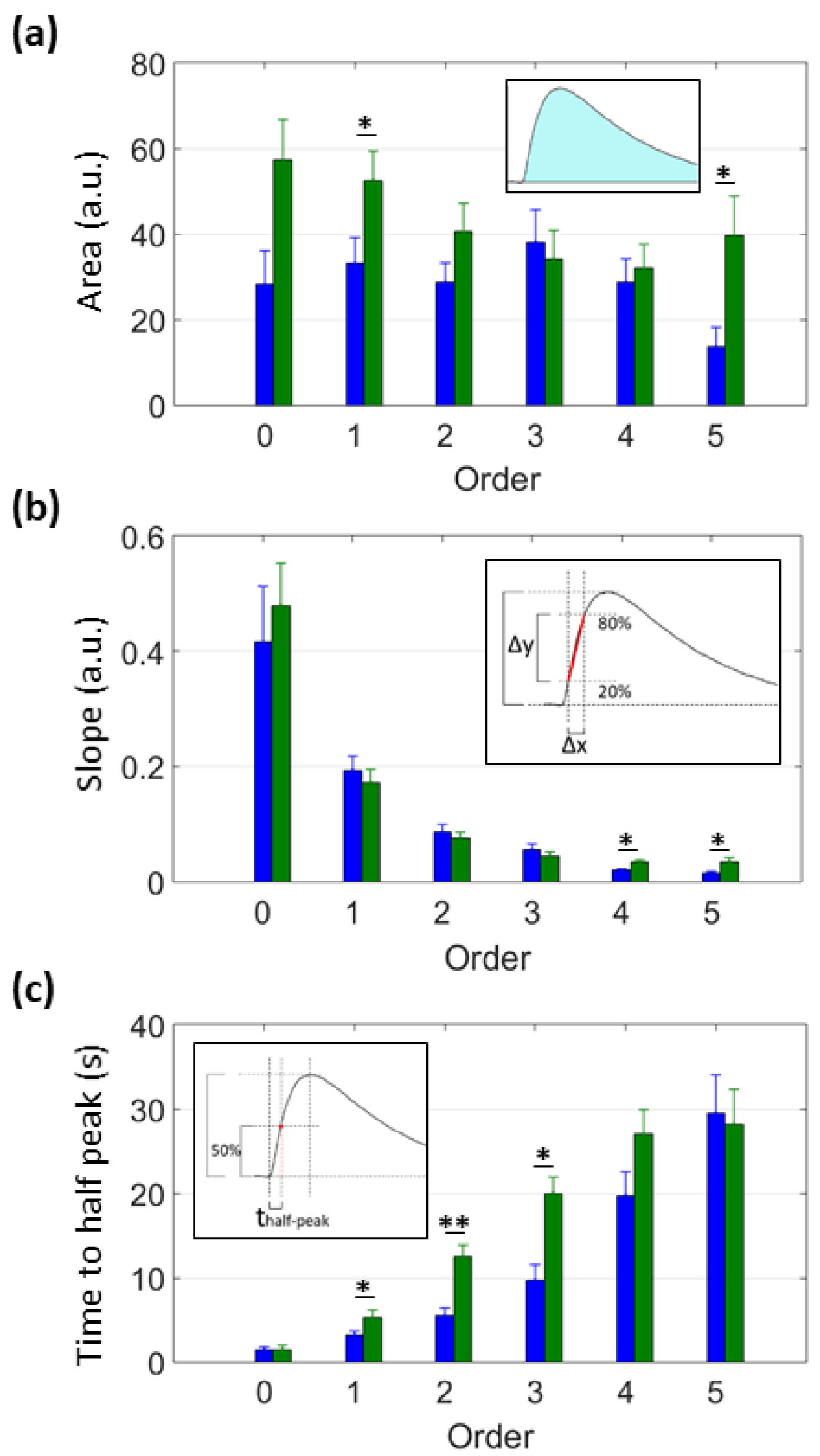

Supplement: Supplementary file 1 [file cells-08-01175-s001.zip › SupplementaryFilesRevised/SupplementaryFigureFiles/FigS4.tif]

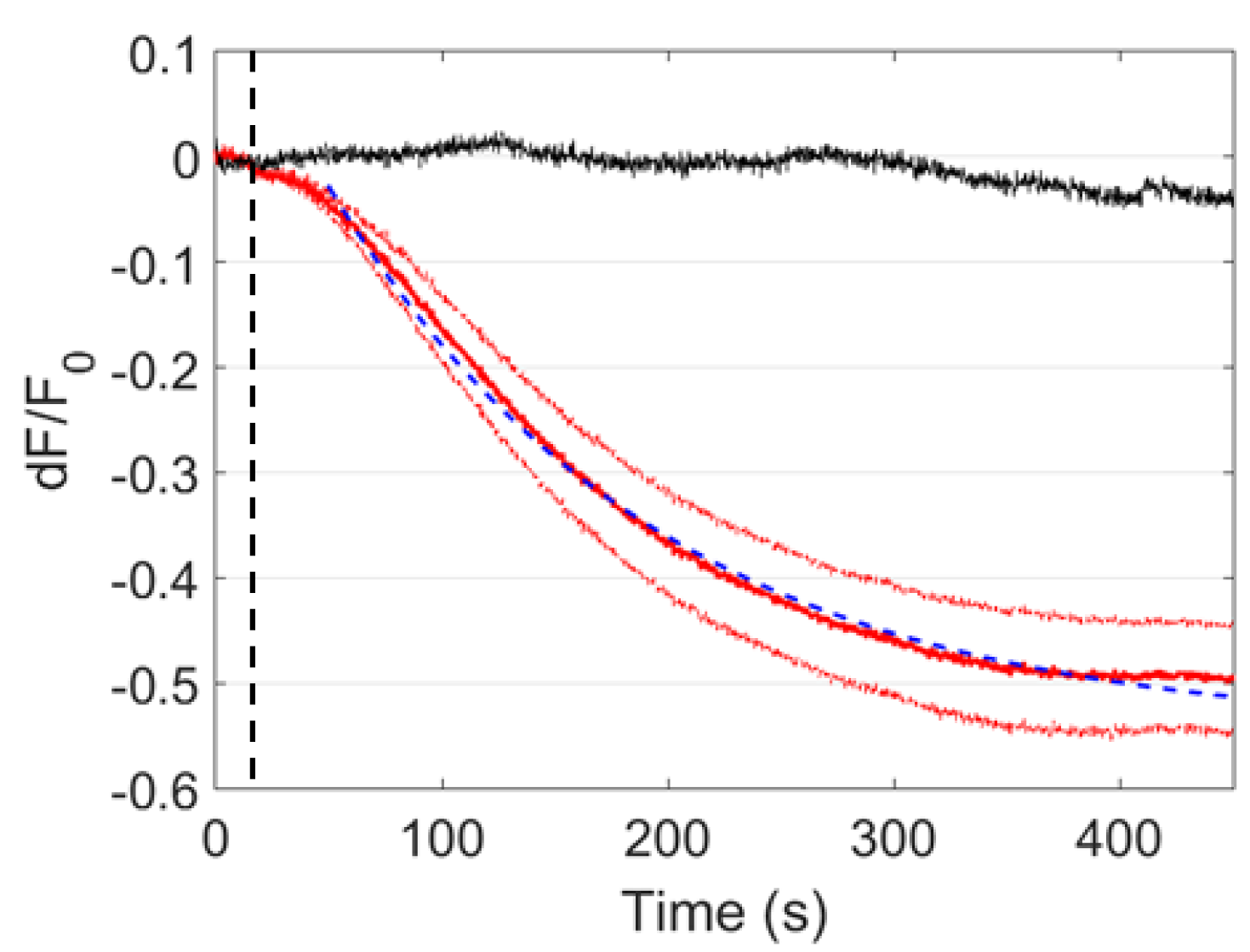

Supplement: Supplementary file 1 [file cells-08-01175-s001.zip › SupplementaryFilesRevised/SupplementaryFigureFiles/FigS5.tif]

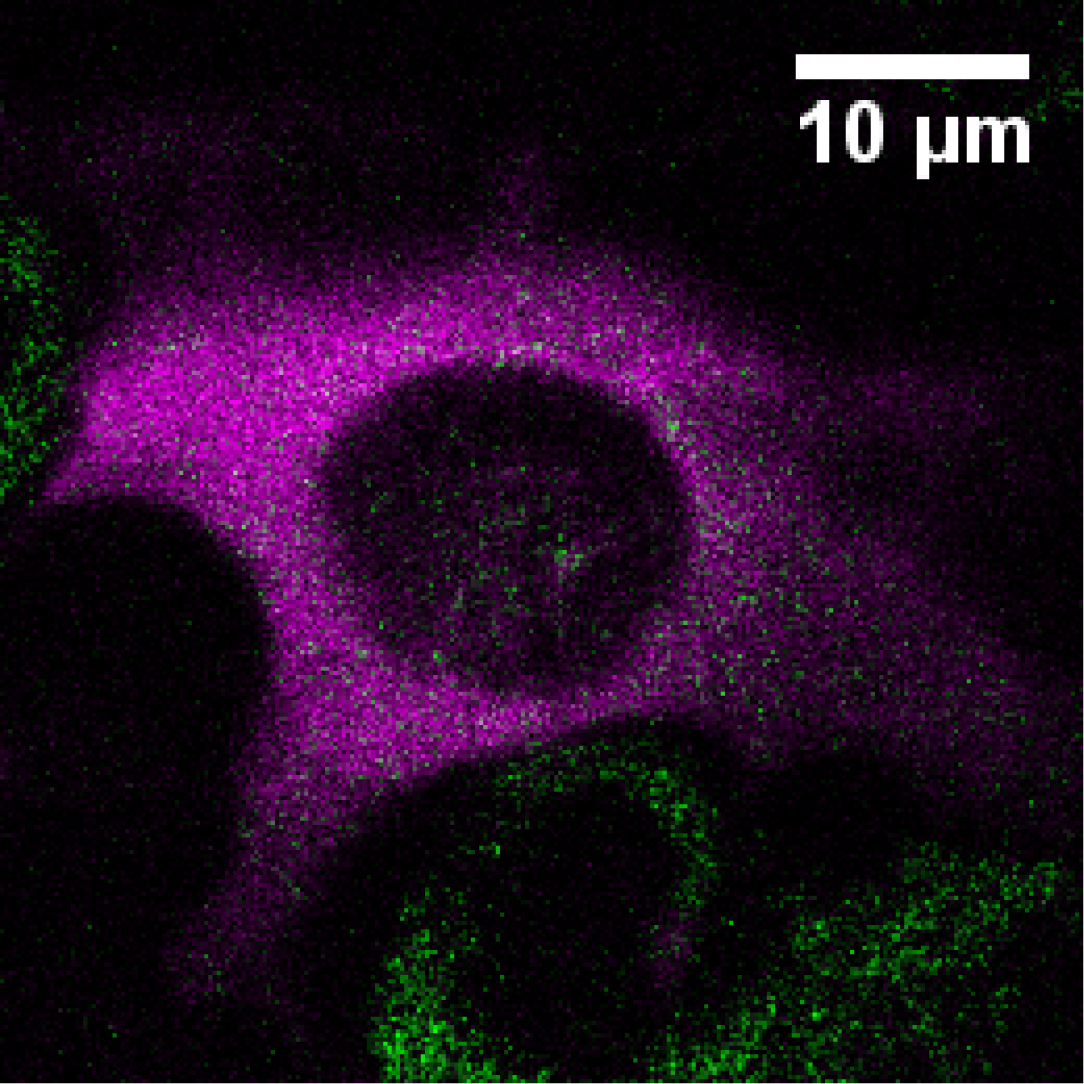

Supplement: Supplementary file 1 [file cells-08-01175-s001.zip › SupplementaryFilesRevised/SupplementaryVideoFiles/VideoV1_first_frame.tif]

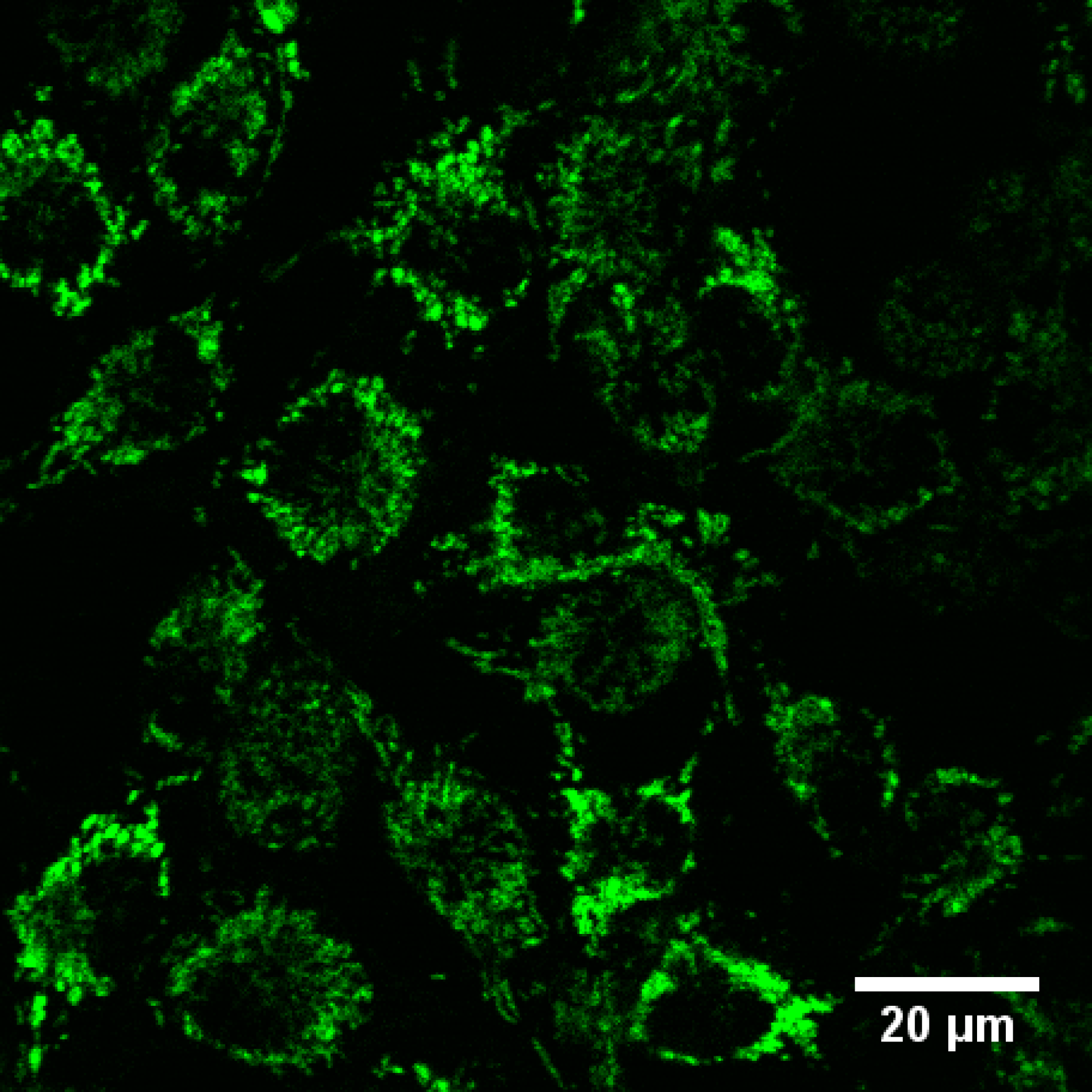

Supplement: Supplementary file 1 [file cells-08-01175-s001.zip › SupplementaryFilesRevised/SupplementaryVideoFiles/VideoV2_first_frame.tif]

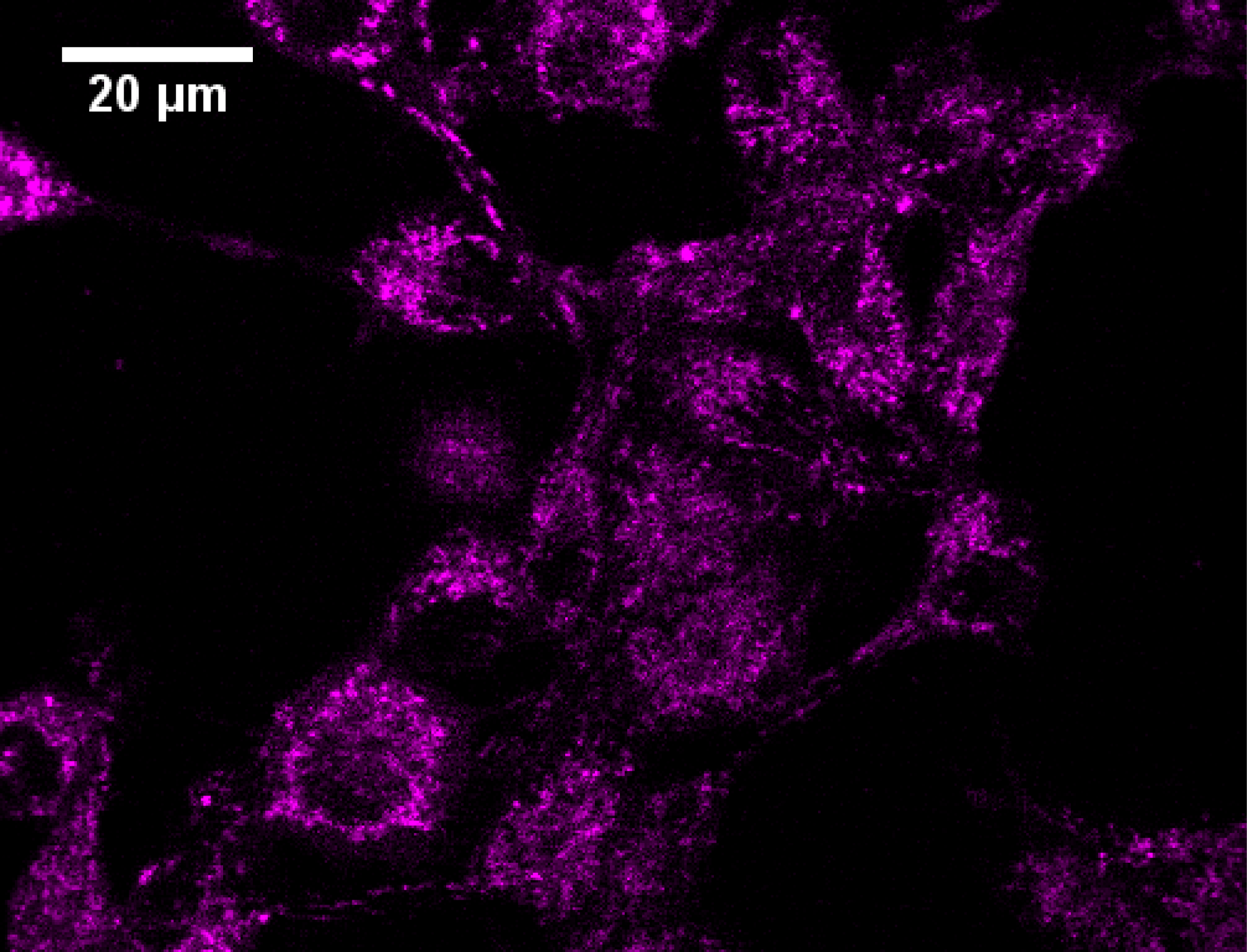

Supplement: Supplementary file 1 [file cells-08-01175-s001.zip › SupplementaryFilesRevised/SupplementaryVideoFiles/VideoV3_first_frame.tif]
